# The High Prevalence of Negative Hepatitis B Surface Antibody (Anti-HBs) among Pregnant Women in Bandung, Indonesia: A Community-Based Study

**DOI:** 10.1155/2020/3414869

**Published:** 2020-10-19

**Authors:** Dolvy Girawan, Raden T. D. Judistiani, Nelly A. Risan, Muhammad B. Bestari, Eka S. Nugraha, Yudith S. Ermaya, Dwi Prasetyo

**Affiliations:** ^1^Division of Gastroenterology and Hepatology, Department of Internal Medicine, Faculty of Medicine, Universitas Padjadjaran, Dr. Hasan Sadikin General Hospital, Bandung 40161, Indonesia; ^2^Centre of Immunology Studies, Faculty of Medicine, Universitas Padjadjaran, Bandung 40161, Indonesia; ^3^Department of Public Health, Faculty of Medicine, Universitas Padjadjaran, Bandung 40161, Indonesia; ^4^Department of Child Health, Faculty of Medicine, Universitas Padjadjaran, Dr. Hasan Sadikin General Hospital, Bandung 40161, Indonesia

## Abstract

**Background:**

Hepatitis B virus (HBV) infection is a disease that creates a high global burden by affecting approximately 3.5% of the total world population. The main transmission of this disease is from mother to child (MTCT). HBV vaccination program was already initiated in Indonesia in 1987. However, after three decades, the HBV infection prevalence stays stagnant. This study aimed to explore the seroprevalence of HBV markers and the attributable risk factors of pregnant women at risk of transmitting HBV to their offspring.

**Method:**

A cross-sectional study was conducted on pregnant women from primary midwifery and obstetric clinics across Bandung, Indonesia, to assess the HBsAg, anti-HBc, and anti-HBs serological markers. Questionnaire-based interviews were used to obtain the sociodemographic determinants. Logistic regression was applied to assess the association of each determinant factor to positive HBsAg or negative anti-HBs as a dependent variable, which was then reported as odds ratios (OR).

**Results:**

A total of 196 subjects were recruited with 12/196 (6.1%) of them were positive HBsAg. After exclusions of those with positive HBsAg and anti-HBc, 24/175 (13.7%) women were isolated as positive anti-HBs, leaving 151/175 (86.3%) women with negative anti-HBs who were susceptible to HBV infection. Low body mass index (BMI) less than 18.5 kg/m^2^ was a risk factor for positive HBsAg with OR = 5.850 (95% CI 1.466-23.34), *p* = 0.012. Nevertheless, no significant determinant factor was associated with negative anti-HBs.

**Conclusion:**

Most pregnant women in Bandung, Indonesia, are susceptible to HBV infection, as marked by the negative anti-HBs status.

## 1. Introduction

It is estimated that 257 million people, or 3.5% of the world population, are living with chronic HBV (hepatitis B virus) infection. The African and Western Pacific account for 68% of HBV infection [1]. Untreated HBV infection leads to cirrhosis and hepatocellular carcinoma as a life-threatening complication, responsible for approximately 96% of hepatitis-related deaths [1, 2]. Indonesia ranks second after India in terms of the country with the highest number of HBV infections in the Asia Pacific region, contributing up to 74% of deaths caused by liver cancer globally [3]. An Indonesian nationwide survey in 2013, the 2013 Indonesian Basic Health Research (IBHR), has reported an HBsAg prevalence of 7.1%. Therefore, the Indonesian HBV infection endemicity is at a moderate level. However, considering the country's total population size of nearly 250 million people, the absolute number of HBV-infected individuals is high [4].

Various attempts have been made to fight HBV infections, including the mass HBV vaccination program for newborns, which dated back from a pilot project in 1987 in Lombok, West Nusa Tenggara Province, followed by the national program of hepatitis B vaccination for all newborns in 1997 and training of health care providers in rural areas. It seems that the progress and accomplishments of HBV control in Indonesia are promising [5]. However, the data in 2013 IBHR presented a surprising fact that the positive HBsAg prevalence among children under five years old was still around 4.2% [6]. Mother to child transmission (MTCT) is reported as the leading cause of HBV infections, although horizontal transmission during the early childhood period might also be the possible cause [1, 2].

Muljono stated that the positive HBsAg prevalence among 69,891 pregnant women across 12 provinces in Indonesia was 2.76% in 2015, with the lowest found in West Sumatra Province (1.6%) and the highest in West Papua Province (8.0%) [7]. These facts indicated that upscaled prevention efforts and more research are needed to stop the transmission by eliminating its causes and to achieve the national HBV elimination target.

The high HBV infection prevalence and low HBV vaccination coverage in Indonesia might be due to the unmet need of vaccination caused by challenges in vaccine supply on the community level, limited transport capacity, and inadequate cold chain capacity. Poor communication among health care professionals regarding who is responsible for HBV prevention and treatment also contributes to this matter [5].

The Government of Indonesia has issued a Decree of the Minister of Health in 2017, which sets a commitment to triple elimination of hepatitis, syphilis, and HIV infection by 2030. The government has implemented a program to screen HBV infections among pregnant women through HBsAg rapid test program [8]. Nonetheless, this screening approach has a drawback in those women who suffer from occult chronic HBV infection or mutant HBV infection could be missed. Consequently, these pregnant women become the potential source for HBV infection transmission to their offspring [9].

So far, no protocols have been developed for anti-HBs testing at any time after the completion of HBV vaccination during childhood and beyond. The assessment of chronic HBV infection and HBV serological status among women in the preconceptional period is actually the best approach to achieve the goal for HBV infection elimination, albeit more expensive [8].

Based on those facts, the Centre of Immunology Studies, Faculty of Medicine, Universitas Padjadjaran, has conducted a study to explore the seroprevalence of HBV markers, including HBsAg, anti-HBc, and anti-HBs, among pregnant women in the community of Bandung city to represent Indonesia. This study emphasized the relation between baseline characteristics, socioeconomic determinants, and anti-HBs status as a long-term evaluation of vaccination coverage. Consequently, the results will provide crucial information to improve the HBV elimination programs among women in child-bearing age in Indonesia.

## 2. Materials and Methods

### 2.1. Study Design

This is a cross-sectional study on pregnant women in the community of Bandung, Indonesia. Questionnaire-based interviews were performed, and blood samples were collected at the same time as the interview by trained research assistants and laboratory technicians.

### 2.2. Study Population and Participant Recruitment

This study was conducted from July 2018 to April 2019 in 27 midwifery clinics and one private obstetric clinic in Bandung. The inclusion criteria were women with singleton near-term pregnancy (35 weeks and/or above) who resided in Bandung as proven by the identity card. The women were given complete information regarding the study objectives, procedures, and all phases of this study during their antenatal care visits or at admission for parturition. The participation in the study was voluntary, and all participants were free to withdraw from the study anytime.

### 2.3. Data Collection Procedure

Data on demographic characteristics and other socioeconomic determinants were obtained through interviews. Approximately 10 ml of blood was sampled from the women's middle cubital vein for complete blood count (CBC) and HBV serological marker (HBsAg, anti-HBs, and anti-HBc) examinations. The remaining serum was stored for other laboratory examinations as clinically indicated or further research purposes. HBV serological markers reported in this article were based on the results of ELISA tests using Siemens ADVIA Centaur® XP immunoassay system, Erlangen, Germany.

### 2.4. Variables

The outcomes were set as a binary variable of HBsAg. The outcome was defined as “positive” if the participant was tested as positive for HBsAg and “negative” if the participant was tested negative for HBsAg. The same definition was also applied to the outcomes of anti-HBc and anti-HBs. The independent variables used in this study were sociodemographic characteristics that included age, weight, height, body mass index (BMI), maternal and spouse employment, cumulative monthly income, maternal education, HBV and tetanus toxoid vaccine history, obstetric history, insurance, ethnicity, and various possible routes for HBV infection such as surgery, tattoo, blood transfusion, injecting drugs, family history of HBV, and previous personal HBV infection history.

### 2.5. Statistical Methods and Data Analysis

Data were analyzed using the Statistical Package for the Social Sciences (SPSS) version 24. The descriptive summary of the HBV serological marker results, i.e., the HBsAg, anti-HBc, and anti-HBs, is presented in Table 1. The outcomes of positive HBsAg and negative anti-HBs were analyzed in relation to the independent variables. Logistic regression was applied to explore the risk factors associated with the results, then reported as the odds ratio (OR) with 95% confidence interval (CI). We considered *p* < 0.05 as significant.

### 2.6. Ethics

Ethical approval was issued by the Health Research Ethical Committee of the Faculty of Medicine, Universitas Padjadjaran. All study subjects had given written informed consent to participate. Consent for publication of the study result without disclosing personal identity was also obtained.

## 3. Results

Participants' baseline characteristics, sociodemographic determinants, and positive or negative HBsAg results are listed in Table 1. The mean age of participants was 27.7 years (SD 6.1), with the majority were in the 25–29 years old group (30.1%). Among these pregnant women, 6.1% were HBsAg positive. Most participants were unemployed (73.0%), and half of the spouses had a part-time job (51.0%) with a cumulative monthly income of less than 250 USD (61.2%). Almost all participants (98.5%) in both positive HBsAg and negative HBsAg groups did not recall having HBV vaccination in the past. Among all determinants, only being underweight was associated with having positive HBsAg (OR = 5.850 with 95% CI 1.466–23.34, *p* = 0.012).

Table 2 shows the combination results of HBV serological markers of all participants and their interpretation, which are classified into eight groups. Subjects with positive HBsAg, positive anti-HBc, but negative anti-HBs accounted for 1.5% of this study population. Meanwhile, only 4.6% had isolated positive HBsAg. However, the triple-negative serological marker finding was predominant in this study (77.0%), while isolated positive anti-HBs accounted for 12.3% of the study population.

Further analyses on the association of baseline and socioeconomic determinants and negative anti-HBs are displayed in Table 3. After the exclusion of positive HBsAg and/or anti-HBc, a total of 175 of 196 subjects were included. Bivariate analysis revealed none of the determinants had significant associations with the negative anti-HBs result.

## 4. Discussions

This was the first seroprevalence study of HBsAg, anti-HBc, and anti-HBs status among pregnant women in Bandung, Indonesia, that aimed at assessing the risk for HBV mother to child transmission (MTCT). The overall prevalence of HBsAg positive was 6.1% in the screened population. This prevalence is comparable to the prevalence among pregnant women in Makassar, Indonesia, which is 6.8% [10]. Despite several wide-scale vaccination campaigns that have been held since 1985, these findings indicate that the Indonesian HBV prevalence remains in the intermediate group according to the WHO criteria, as the prevalence is between 2 and 8% [11]. Other neigbouring Southeast Asian countries also present an HBV infection prevalence that ranges from moderate (2–8%), such as in Thailand (3%), to high (>8%), such as the Philippines (16%) [3].

This study revealed that approximately 78.5% of these women were between 20 and 34 years old. Nearly all participants in the positive HBsAg group had at least one or more children, with half of them might be very likely to have another pregnancy some time in the future. Around 61.2% of women came from a low-income family (less than 250 USD per month), which might be a constraint for getting HBV vaccination although the majority of these women (83.7%) had completed senior high school.

Routine HBV screening during pregnancy using the maternal HBsAg test has been suggested by the Society for Maternal-Fetal Medicine [12]. New evidence from Indonesia has proven that the HBsAg level provides an excellent viral load predictor in HBeAg-positive pregnant women, but not in HBeAg-negative pregnant women [10]. Cautions should be applied when assessing pregnant women who are in the immune-tolerant phase of chronic HBV infection with high-level viremia but with typical physical or laboratory results. This would, unfortunately, lead to the lack of awareness of their HBsAg-positive status and increase the risk of transmitting the HBV virus to their offspring [10].

A small number of pregnant women in this study had personal or familial behaviour risk factors for HBV infection. Still, those risk factors seem to be unrelated to HBV infection, as marked by positive HBsAg. Almost all of the women (98.5%) did not recall having HBV vaccination. In comparison, women who did not remember the tetanus toxoid (TT) vaccination was only 43.4% in accordance with Table 1). This might not indicate the real condition, and reconfirmation was not feasible due to poor personal records.

Table 2 shows that twelve women (6.1%) were positive HBsAg. Three other women (1.5%) had a combination of positive anti-HBc and negative HBsAg and anti-HBs, reflecting the possibility that they might have occult HBV infections. Further assessment is needed to confirm the diagnosis of HBV among these women. The health impact of HBV infection increases over time as individuals with chronic infection age and develop HBV-related complications. Up to 40% of individuals with chronic HBV infection progress to cirrhosis, liver failure, and hepatocellular carcinoma [13].

As presented in Table 3, a large proportion of subjects (172 of 175 subjects, 98.3%) might not have any HBV vaccination in the past or do not recall past HBV vaccination. This fact is in contrast with the previous report stating that the average HBV vaccination coverage in West Java Province, including Bandung, is as high as 80% [6]. The findings of this study demonstrate that only 24 (12.3%) women had positive anti-HBs, while 151 (77.0%) were negative anti-HBs. Those women with negative anti-HBs were at-risk or susceptible to HBV infection. Therefore, keeping good vaccination records of every pregnant woman and newborn should be further encouraged. The recorded data should contain information on the type of vaccine used and the time of administration. The Ministry of Health has actually provided a book for mother and child health record known as “Buku Kesehatan Ibu dan Anak” or “Buku KIA” to be used to record data on individual mother and child. This book provides spaces for recording vaccination and is distributed for free and routinely used in primary health centres. It should be encouraged that this book should be kept as an important documentation for life.

This study has identified various baseline characteristics and socioeconomic factors that may be associated with the negative anti-HBs result (see Table 3). Identification of risk factors is essential to inform the stakeholders of the HBV prevention program. Although the statistical analysis of the independent variables against the anti-HBs status does not result in significant results in this study, there are some factors that might be associated with negative anti-HBs, including low income (less than 250 USD per month), unemployment, lack of insurance ownership, and unknown HBV or TT vaccine history. Additionally, in countries with perinatal HBV programs like Indonesia, all infants should receive three scheduled doses of HBV vaccination [5]. The vaccination at 0, 1, and 6 months should attain a positive anti-HBs value of more than 10 mIU/mL to be able to provide adequate protection against HBV infection [9, 14]. It would be best to check for the seroconversion of anti-HBs in these infants.

So far, there has been no protocol available on HBV booster vaccination for women in reproductive age. The real challenge is reflected from 151 of 175 (86.3%) women who are susceptible to HBV infection because of their negative HBsAg, anti-HBc, and anti-HBs status (see Table 2). Two possible explanations are they never have HBV vaccination before and they might be in the nonresponder group [9].

HBV vaccination is indicated in adults who are prone to acquiring HBV infection from frequent exposure to HBV-infected individuals. Vaccination against HBV during pregnancy is safe, and the immunogenicity would be efficient for the passive transfer of maternal antibody to newborns. No significant adverse reaction has been reported in HBV-vaccinated pregnant women as compared to those without vaccination [14].

Recommendations have been made on emergency HBV immunization in high-risk pregnant populations. HBV vaccination, with or without HBV immunoglobulin, is recommended for postexposure prophylaxis to prevent HBV infection. High risk is defined as having a sex partner with positive HBsAg, promiscuity, presence of sexually transmitted disease, and injecting drug behaviour [14].

In practice, the HBV vaccine can be administered to women in 3 doses over a period of 6 months before, during, and after pregnancy, despite that FDA classifies HBV vaccine as a C-level drug for pregnant women. No conclusion on the advisability of HBV vaccination among pregnant women population could be drawn from the Global Advisory Committee on Vaccine Safety, and the rarity of evidence of HBV vaccination in pregnant women would be a real challenge for its application [15, 16].

This study reported a low anti-HBs positive rate in pregnant women (12.3%). This should raise an awareness that the HBV vaccination should be extended to women of child-bearing age. The World Health Organization (WHO) called for the elimination of HBV infection as a public health threat by 2030 and to reduce new chronic infections by 90% and mortality by 65% in 2020. The prevention of vertical transmission from mother-to-child and prevention of infant and childhood infection are a cornerstone of the global strategy and recommended for all countries regardless of their HBV endemicity [2]. The best practice would be HBV vaccination at birth and early infancy. A mathematical model has demonstrated that an HBV vaccine coverage of 90% will prevent 84% of HBV-related deaths worldwide [2].

Pregnant women are considered immunocompromised individuals who experience a decrease in antibody titers [17]. An administration of additional vaccine booster before pregnancy may be suggested. HBV vaccine booster triggers the increase in protective antibody levels for 10 to 30 years [9]. Further studies are needed to solve the problem in nonresponder individuals and also to prove the safety and efficacy of HBV vaccination in pregnant women with negative anti-HBs.

## 5. Conclusions

This study shows that most pregnant women in Bandung, Indonesia, are negative anti-HBs. Therefore, they are not protected from hepatitis B infection. Based on these findings, it is recommended that HBV vaccination is provided to all pregnant women.

## Figures and Tables

**Table 1:** Participant characteristics and HBsAg status.

	All participants	HBsAg positive	HBsAg negative	OR (95% CI)	*p*
Overall, *n* (%)	196 (100)	12 (6.1)	184(93.9)		
Age					
Mean year (SD)	27.7 (6.1)	27.1 (5.2)	27.7 (6.2)		
Distribution, *n* (%)					
13–20	10 (5.1)	1 (0.5)	9 (4.6)	1	
20–24	51 (26.0)	2 (1.0)	49 (25.0)	2.722 (0.223-33.27)	0.433
25–29	59 (30.1)	5 (2.6)	54 (27.6)	1.200 (0.125-11.50)	0.874
30–34	44 (22.4)	3 (1.5)	41 (20.9)	1.519 (0.141-16.32)	0.730
>35	32 (16.3)	1 (0.5)	31 (15.8)	3.444 (0.195-60.71)	0.398
Bodyweight					
Mean kg (SD)	53.6 (9.7)	56.3 (8.9)	53.4 (9.8)		
Height					
Median cm (IQR)	155.0 (6.0)	158.0 (9.0)	155.0 (6.0)		
BMI					
Mean kg/m^2^ (SD)	22.3 (3.9)	23.1 (3.7)	22.3 (3.9)		
Distribution, *n* (%)					
<18.5	30 (15.3)	5 (2.6)	25 (12.8)	1	
18.5–24.9	121 (61.8)	4 (2.0)	117 (59.7)	5.850 (1.466-23.34)	*0.012* ^§^
25–29.9	34 (17.3)	3 (1.5)	31 (15.8)	2.067 (0.450-9.499)	0.351
≥30	11 (5.6)	0 (0)	11 (5.6)	N/A	
Maternal employment, *n* (%)					
Unemployed	143 (73.0)	10 (5.1)	133 (67.9)	1	
Fulltime	26 (13.3)	1 (0.5)	25 (12.8)	1.880 (0.230-15.34)	0.556
Part time	27 (13.8)	1 (0.5)	26 (13.3)	1.955 (0.240-15.93)	0.531
Spouse employment, *n* (%)					
Unemployed	5 (2.6)	0 (0)	5 (2.6)	N/A	
Fulltime	91 (46.4)	6 (3.1)	85 (43.4)	1	
Part time	100 (51.0)	6 (3.1)	94 (48.0)	1.106 (0.344-3.560)	0.866
Family income (monthly)^∗^, *n* (%)					
<250 USD	120 (61.2)	8 (4.1)	112 (57.1)	1	
250–499 USD	59 (30.2)	4 (2.0)	55 (28.1)	0.982 (0.283-3.404)	0.977
500–999 USD	14 (7.1)	0 (0)	14 (7.1)	N/A	
>1000 USD	3 (1.5)	0 (0)	3 (1.5)	N/A	
Education, *n* (%)					
Preschool	1 (0.5)	0 (0)	1 (0.5)	N/A	
Elementary school	8 (4.1)	1 (0.5)	7 (3.6)	1	
Intermediate school	23 (11.7)	3 (1.5)	20 (10.2)	0.952 (0.085-10.72)	0.968
High school	98 (50.0)	5 (2.6)	93 (47.4)	2.657 (0.272-25.98)	0.401
Graduate	64 (32.7)	3 (1.5)	61 (31.1)	2.905 (0.265-31.84)	0.383
Postgraduate	2 (1.0)	0 (0)	2 (1.0)	N/A	
HBV vaccine frequency history, *n* (%)					
Unknown	85 (43.4)	5 (2.6)	80 (40.8)	1	
None	108 (55.1)	7 (3.6)	101 (51.5)	0.902 (0.276-2.948)	0.864
1-3	2 (1.0)	0 (0)	2 (1.0)	N/A	
4	1 (0.5)	0 (0)	1 (0.5)	N/A	
TT vaccine frequency history, *n* (%)					
None	85 (43.4)	5 (2.6)	80 (40.8)	1	
1-2	92 (46.9)	6 (3.1)	86 (43.9)	0.788 (0.218-2.840)	0.715
≥3	19 (9.7)	1 (0.5)	18 (9.2)	0.563(0.059-5.363)	0.617
Gravidae, *n* (%)					
1	72 (36.7)	5 (2.6)	67 (34.2)	1	
2-3	100 (51.0)	7 (3.6)	93 (47.4)	0.991 (0.302-3.259)	0.989
≥4	24 (12.2)	0 (0)	24 (12.2)	N/A	
Parturien, *n* (%)					
0	77 (39.2)	5 (2.6)	72 (36.7)	1	
1-2	103 (52.6)	7 (3.6)	96 (49.0)	0.952 (0.290-3.123)	0.936
≥3	16 (8.2)	0 (0)	16 (8.2)	N/A	
Abortus, *n* (%)					
0	165 (84.2)	11 (5.6)	154 (78.6)	1	
1-2	30 (15.3)	1 (0.5)	29 (14.8)	2.071 (0.257–16.67)	0.494
≥3	1 (0.5)	0 (0)	1 (0.5)	N/A	
Insurance, *n* (%)					
BPJS^∗∗^	53 (27.0)	2 (1.0)	133 (67.9)	1	
None	143 (73.0)	10 (5.1)	51 (26.0)	1.917 (0.406-9.052)	0.411
Maternal ethnicity, *n* (%)					
Sundanese	184 (93.9)	12 (6.1)	172 (87.8)	N/A	
Javanese	8 (4.1)	0 (0)	8 (4.1)		
Batak	1 (0.5)	0 (0)	1 (0.5)		
Malay	1 (0.5)	0 (0)	1 (0.5)		
Others	2 (1.0)	0 (0)	2 (1.0)		
Possible route of HBV infection, yes answered, *n* (%)					
Ever had hepatitis history	0 (0)	0 (0)	0 (0)	N/A	
Ever had surgery	6 (3.1)	0 (0)	6 (3.1)		
Mother's tattoo	2 (1.0)	0 (0)	2 (1.0)		
Spouse's tattoo	3 (1.5)	0 (0)	3 (1.5)		
Ever had a blood transfusion	2 (1.0)	0 (0)	2 (1.0)		
Spouse's blood transfusion history	0 (0)	0 (0)	0 (0)		
Hepatitis history from a spouse	1 (0.5)	0 (0)	1 (0.5)		
Hepatitis history from family^∗∗∗^	1 (0.5)	0 (0)	1 (0.5)		
Mother history of IDU	0 (0)	0 (0)	0 (0)		
Spouse history of IDU	0 (0)	0 (0)	0 (0)		

^§^Significant value. ^∗^Cumulative income rounded and converted from Indonesia Rupiah (IDR) to US Dollar (USD), ^∗∗^BPJS = Indonesian National Insurance, ^∗∗∗^Mother (first ascend) or siblings. OR: odds ratio; CI: confidence interval, N/A: not applicable; TT: tetanus toxoid; IDU: injecting drug user.

**Table 2:** Hepatitis B infection serological markers.

HBsAg	Anti-HBc	Anti-HBs	All, *n* (%)	Born before 1990, *n* (%)	Born after 1990, *n* (%)
**+**	**+**	**+**	0 (0)	0 (0)	0 (0)
**+**	**+**	−	3 (1.5)	1 (0.5)	2 (1.0)
**+**	−	**+**	0 (0)	0 (0)	0 (0)
**+**	−	−	9 (4.6)	3 (1.5)	6 (3.0)
−	**+**	**+**	6 (3.1)	4 (2.0)	2 (1.0)
−	**+**	−	3 (1.5)	3 (1.5)	0 (0)
−	−	**+**	24 (12.3)	12 (6.1)	12 (6.1)
−	−	−	151 (77.0)	69 (35.2)	82 (41.8)
Total			196 (100)	92 (46.9)	104 (53.1)

**Table 3:** Analyses of anti-HBs-negative subjects after excluding HBsAg- and Anti-HBc-positive subjects.

	Frequency	Negative anti-HBs	OR (95% CI)	*p*
Overall, *n* (%)	175 (100)	151 (86.3)		
Age distribution, *n* (%)				
13–20	9 (5.1)	7 (4.0)	1	
20–24	49 (28.0)	44 (25.1)	0.398 (0.064–2.463)	0.322
25–29	51 (29.1)	45 (25.7)	0.467 (0.078–2.788)	0.403
30–34	35 (20.0)	30 (17.1)	0.583 (0.093–3.653)	0.565
>35	31 (17.7)	25 (14.3)	0.840 (0.138–5.115)	0.850
BMI distribution, *n* (%)				
<18.5	25 (14.3)	21 (12.0)	1	
18.5–24.9	110 (62.9)	96 (54.9)	0.766 (0.229–2.561)	0.665
25–29.9	30 (17.1)	26 (14.9)	0.808 (0.180–3.622)	0.780
≥30	10 (5.7)	8 (4.6)	1.312 (0.200–8.624)	0.777
Maternal employment, *n* (%)				
Unemployed	128 (73.1)	114 (65.1)	1	
Fulltime	24 (13.7)	19 (10.9)	2.143 (0.692–6.638)	0.186
Part time	23 (13.1)	18 (10.3)	2.262 (0.727–7.042)	0.159
Spouse employment, *n* (%)				
Unemployed	5 (2.9)	3 (1.7)	1	
Fulltime	82 (46.9)	70 (40.0)	0.257 (0.039–1.704)	0.257
Part time	88 (50.3)	78 (44.6)	0.192 (0.029–1.294)	0.192
Family income (monthly)^∗^, *n* (%)				
<250 USD	106 (60.6)	90 (51.4)	1	
250–499 USD	54 (30.9)	48 (27.4)	0.703 (0.258–1.914)	0.491
500–999 USD	12 (6.9)	11 (6.3)	0.511 (0.062–4.239)	0.534
>1000 USD	3 (1.7)	2 (1.1)	2.813 (0.241–32.87)	0.410
Education, *n* (%)				
Preschool	1 (0.6)	1 (0.6)	N/A	
Elementary school	6 (3.4)	5 (2.9)	1	
Intermediate school	19 (10.9)	16 (9.1)	0.938 (0.079–11.15)	0.959
High school	90 (51.4)	77 (44.0)	0.844 (0.091–7.819)	0.881
Graduate	57 (32.6)	51 (29.1)	0.588 (0.059–5.912)	0.652
Postgraduate	2 (1.1)	1 (0.6)	5.0 (0.150–166.6)	0.368
HBV vaccine frequency history, *n* (%)				
Unknown	75 (42.9)	61 (34.9)	1	
None	97 (55.4)	87 (49.7)	1.997 (0.832-4.780)	0.121
1-3	2 (1.1)	2 (1.1)	N/A	
4	1 (0.6)	1 (0.6)	N/A	
Insurance, *n* (%)				
BPJS^∗∗^	48 (27.4)	44 (25.1)	1	
None	127 (72.6)	107 (61.1)	0.486 (0.157–1.505)	0.211
Mother ethnicity, *n* (%)				
Sundanese	165 (94.3)	142 (81.1)	1	
Javanese	7 (4.0)	6 (3.4)	1.029 (0.118–8.944)	0.979
Batak	1 (0.6)	1 (0.6)	N/A	
Malay	1 (0.6)	1 (0.6)	N/A	
Others	1 (0.6)	1 (0.6)	N/A	

^∗^Cumulative income rounded and converted from Indonesia Rupiah (IDR) to US Dollar (USD), ^∗∗^BPJS = Indonesian National Insurance, OR: odds ratio; CI: confident interval; N/A: not applicable.

## Data Availability

The datasets generated during and/or analysed during the current study are available from the corresponding author on reasonable request. Correspondence or request should be addressed to the Division of Gastroenterology and Hepatology, Department of Internal Medicine Hasan Sadikin General Hospital, Universitas Padjadjaran. Address: Jalan Pasteur 38 Bandung, 40161, Indonesia. E-mail: eka.surya@unpad.ac.id.
